# Preoperative dietitian-led Very Low Calorie Diet (VLCD) Clinic for adults living with obesity undergoing gynaecology, laparoscopic cholecystectomy and hernia repair procedures: a pilot parallel randomised controlled trial

**DOI:** 10.1017/S0007114524000114

**Published:** 2024-04-28

**Authors:** Sally B. Griffin, Michelle A. Palmer, Esben Strodl, Rainbow Lai, Teong L. Chuah, Matthew J. Burstow, Lynda J. Ross

**Affiliations:** 1 Department of Nutrition & Dietetics, Logan Hospital, Meadowbrook, QLD, Australia; 2 School of Exercise and Nutrition Sciences, Queensland University of Technology, Brisbane, QLD, Australia; 3 School of Psychology and Counselling, Queensland University of Technology, Brisbane, QLD, Australia; 4 Surgical and Critical Care Services, Logan Hospital, Meadowbrook, QLD, Australia; 5 Department of Surgery, Mater Hospital, South Brisbane, QLD, Australia; 6 Mayne Academy of Surgery, Faculty of Medicine, University of Queensland, St Lucia, QLD, Australia; 7 School of Medicine and Dentistry, Griffith University, Gold Coast, QLD, Australia

**Keywords:** VLCD, VLED, Dietitian, Diet, Weight loss, Obesity, Prehabilitation

## Abstract

Obesity can increase the risk of postoperative complications. Despite increased demand for patients living with obesity to lose weight prior to common surgical procedures, the impact of intentional weight loss on surgical outcomes is largely unknown. We aimed to conduct a pilot study to assess the feasibility of a full-scale randomised controlled trial (RCT) to examine the effect of preoperative dietitian-led Very Low Calorie Diet (VLCD) Clinic on surgical outcomes in gynaecology and general surgeries. Between August 2021 and January 2023, a convenience sample of adults living with obesity (BMI ≥ 30 kg/m^2^) awaiting gynaecology, laparoscopic cholecystectomy and ventral hernia repair procedures were randomised to dietitian-led VLCD (800–1000 kcal using meal replacements and allowed foods), or control (no dietary intervention), 2–12 weeks preoperatively. Primary outcome was feasibility (recruitment, adherence, safety, attendance, acceptability and quality of life (QoL)). Secondary outcomes were anthropometry and 30-d postoperative outcomes. Outcomes were analysed as intention-to-treat. Fifty-one participants were recruited (*n* 23 VLCD, *n* 28 control), mean 48 (sd 13) years, 86 % female, and mean BMI 35·8 (sd 4·6) kg/m^2^. Recruitment was disrupted by COVID-19, but other thresholds for feasibility were met for VLCD group: high adherence without unfavourable body composition change, high acceptability, improved pre/post QoL (22·1 ± 15 points, < 0·001), with greater reductions in weight (–5·5 kg VLCD *v*. −0·9 kg control, *P* < 0·05) waist circumference (–6·6 cm VLCD *v*. +0·6 control, *P* < 0·05) and fewer 30-d complications (*n* 4/21) than controls (*n* 8/22) (*P* > 0·05). The RCT study design was deemed feasible in a public hospital setting. The dietitian-led VLCD resulted in significant weight loss and waist circumference reduction compared with a control group, without unfavourable body composition change and improved QoL.

The number of adults living with obesity has tripled worldwide since 1975 and continues to rise in the Western world^(1)^. Abdominal adiposity and hepatosteatosis can hinder port placement and operative exposure and increase technical difficulty of gynaecological and general surgical procedures^(2,3)^. This can increase the length of procedures and complication risk, attributing major costs to surgical care^(4)^. Therefore, with increased demand for elective surgery, the preoperative period is now considered a critical time to prehabilitate patients living with obesity.

A very low calorie diet (VLCD), defined as ≤ 800 kcal/d^(5)^, is currently the most effective non-pharmacological, non-surgical approach to weight loss for adults living with obesity^(6,7)^, with guidelines suggesting VLCD should be considered for adults who require weight loss prior to surgery^(8,9)^. Preoperative VLCD is now routine for patients who undergo bariatric surgery, to reduce liver size to improve surgical access, based on established evidence in reducing 30-d postoperative complications^(10,11)^. It is vital for VLCD to be prescribed and monitored by a dietitian for nutritional adequacy to reduce the risk of lean body mass (LBM) loss^(12)^. LBM loss in the preoperative period can compound the protein catabolism which occurs during surgery, delaying wound healing, compromising immune function, and diminishing muscle strength and ability to mobilise postoperatively, thereby increasing the risk of postoperative complications^(13,14)^.

The efficacy of a preoperative dietitian-led VLCD intervention delivered via a ‘VLCD Clinic’ designed specifically for this purpose was recently evaluated for adults living with obesity undergoing a range of common elective surgical procedures^(15)^. The same diet prescription as the current study was used: a diet using one, two or three commercial VLCD meal replacement products and additional protein sources to meet individual protein requirements, resulting in intakes of 800–920 kcal per d for most participants. The cohort of seventy-eight patients demonstrated clinically significant weight loss and surgeons reported easier (83 %, *n* 10/12) and shorter (75 %, *n* 9/12) procedures. There are few randomised controlled trials (RCT) that have examined VLCD prior to non-bariatric surgeries. However, published studies have found that short-term very low/low calorie diets (≤ 900 kcal/d) of 1–3 weeks can result in reduction in blood loss for liver resection^(16)^ and gastrectomy^(17)^ (–27 to –411 ml, *P* < 0·05), for laparoscopic cholecystectomy, reduction of 6 minutes in operating time (*P* < 0·05) and reduced difficulty of procedure (*P* < 0·05)^(18)^. Hernia repair has been examined in one RCT, which utilised a general healthy eating intervention, but between-group difference in weight loss was not statistically or clinically significant and the effect on surgical outcomes could not be established^(19)^. Gynaecology surgery has not been explored, despite potential major benefit, as most procedures require laparoscopic access which is hindered by abdominal adiposity^(20)^.

This pilot RCT aimed to inform the design of a full-scale RCT to evaluate the effect of preoperative dietitian-led VLCD Clinic intervention on surgical outcomes for patients living with obesity awaiting elective gynaecology, ventral hernia repair and laparoscopic cholecystectomy procedures. Feasibility was determined via measures of recruitment, VLCD adherence, safety, attendance, acceptability and health-related quality of life (QoL). The study also aimed to gain preliminary data on a range of secondary outcomes: patient characteristics, anthropometric data (weight, BMI, waist, hip and neck circumference, waist:hip ratio, and fat mass), duration of intervention, duration and/or intensity of structured exercise (both groups) or engagement in other dietary interventions (control group), QoL measures using health state index, and a range of surgical outcomes.

## Methods

### Study design and recruitment

This single-centre, prospective, parallel RCT had a 1:1 allocation ratio allocated in blocks of three elective surgery types (laparoscopic cholecystectomy, ventral hernia repair and major gynaecology procedures). It was conducted at a large public hospital in Southeast Queensland, Australia, adhering to CONSORT 2010 Guidelines. Recruitment took place between August 2021 and January 2023, with a 3-month COVID-19 hiatus. This study was conducted according to the guidelines laid down in the Declaration of Helsinki, and all procedures involving human patients were approved by the Metro South Health Human Research Ethics Committee (HREC/2021/QMS/68901) and the University Human Research Ethics Committee (QUT 2021000155). Written informed consent was obtained from all patients. The study procedures and protocol were approved and prospectively registered with the Australia New Zealand Clinical Trials Registry (ACTRN12621000084886p).

Eligible patients were adults with a BMI ≥ 30 kg/m^2^ at the time of their surgical booking for elective laparoscopic cholecystectomy, ventral hernia repair (excluding inguinal hernia) and major gynaecology procedures. Persons with any of the following were considered ineligible: type 1 diabetes, metastatic cancer, liver/kidney failure, acute CVD, pregnant/breast-feeding, preparing/undergoing *in vitro* fertilisation, overt psychosis/severe mental impairment, currently being treated in the existing on-site VLCD Clinic, malnourished/at risk (using the Subjective Global Assessment^(21)^ at initial appointment) or medically unsuitable for VLCD per treating surgeon. If participants’ surgical care was transferred to a private facility after they had consented, they were still included in the study.

Potential participants were screened from surgical waiting lists by the principal investigator. Those with BMI ≥ 30 kg/m^2^ were sent a letter regarding potential eligibility and then telephoned and screened using remaining eligibility criteria. All eligible participants were asked to attend an initial session to undertake formal recruitment, randomisation and baseline data collection. Participants were not provided with weight loss targets to achieve prior to surgery.

Participants were randomly allocated to undertake either VLCD group (intervention) or standard care group (control) by a researcher who was blinded and not involved in recruitment, using simple randomisation via random number generator in EXCEL, version 2212 (Microsoft Corp.). Allocation was concealed until after informed consent was gained. The ‘unhide’ function in EXCEL was used to reveal group allocation. Participants and the treating dietitian were not blinded to group allocation given the nature of the intervention. Blinding of group allocation to the surgeon was not possible given the visibility of dietitian documentation in medical records. The researcher analysing the data was not blinded to group allocations.

### Very low calorie diet group conditions

Fortnightly dietitian appointments were scheduled with a single treating dietitian from consent until surgery. The number of dietitian appointments varied between individual participants depending on the type of surgery. For laparoscopic cholecystectomy participants, their first appointment was scheduled 2 weeks prior to their surgery (where able), which aligned with the timeline required to elicit optimal liver volume reduction^(17,18)^. Gynaecology and hernia repair participants commenced VLCD between 3 and 12 weeks prior to surgery, to elicit more significant visceral fat loss beyond liver volume reduction (which specifically benefits laparoscopic cholecystectomy).

Participants aged < 65 years were recommended to follow ‘Phase 1 VLCD’ which comprised three VLCD meal replacements daily (800 kcal/d, about 3400 kJ/d), while participants aged ≥ 65 years were recommended to follow ‘Phase 2 VLCD’ due to decreased metabolic and physiologic adaptations in response to intensive VLCD^(22)^. Phase 2 comprised two VLCD meal replacements daily (about 900 kcal/d and about 3800 kJ/d) and included two portions of carbohydrate containing whole foods to replace the third VLCD meal replacement product (e.g. 15 g carbohydrate portion = 1 apple). Regardless of the phase prescribed, all participants were asked to consume ≥ 2 cups of low-starch vegetables and ≥ 2 litres of calorie-free fluids daily. Additional protein-rich food(s) were also prescribed, if required, to meet individual protein requirements (based on 0·8–1 g protein/kg of adjusted ideal body weight/d). Participants could choose from a range of commercial VLCD meal replacement products (shakes, bars, soups or desserts) from Optifast^®^ (Nestle Health) or Optislim^®^ (OptiPharm), purchased at their own expense. The nutritional breakdown of these products is provided in online Supplementary Table S1. Although the kcal intake of some participants may have exceeded the calorie-defined description of a VLCD (≤ 800 kcal/d)^(5)^, results from the previous evaluation study^(15)^ showed > 90 % of participants follow the most intensive prescription (Phase 1 – 800–920 kcal/d) when treated with this dietetic intervention. Therefore, the term ‘VLCD’ was used to describe the intervention as a whole as it was more comparable to a VLCD than a low-calorie diet, defined as 800–1600 kcal per d, and more often based on food alone^(5)^.

At each fortnightly appointment, the dietitian utilised the Nutrition Care Process^(23)^ to guide treatment. Where necessary to aid adherence, and in collaboration with the participant, the dietitian could recommend changes to the dietary plan, by way of meal timings, food/product preferences and additions of suitable foods. The dietitian encouraged adherence to Phase 1 if it was achievable and appropriate (age < 65 years) for the participant, but transition to Phase 2 was supported if Phase 1 became challenging, consistent with supportive dietetic care. Appropriate structured physical activity was encouraged based on participants’ physical ability and in line with Australian government recommendations (e.g. 2·5–5 h of moderate-intensity physical activity per week for adults 18–64 years plus muscle strengthening activities). Participants were asked at each appointment about physical activity and continuation/increase was encouraged, and practical strategies were suggested (e.g. choosing enjoyable outdoor weekend activities, increasing incidental activity and utilisation of mobile apps for workouts). Education on progression to healthy eating after surgery was provided. Written resources were provided: a weight record, local low-cost exercise programme, dietary guidelines and a recipe book.

### Control group conditions

Control conditions replicated standard care for elective surgery patients at the research site. Participants were not provided dietary advice and were neither encouraged nor asked to discontinue any self-initiated dietary changes or increase in physical activity.

Participants in both groups were provided with an incentive ($10AUD gift voucher redeemable at well-known grocery/homeware stores) if they attended their two sessions for data collection.

### Data collection

All participants attended two sessions, where the same data were collected: the first at the initial recruitment session (baseline), and the second on, or as close to, surgery day as possible (post). Surgical outcomes were collected from electronic medical records up to 30 d post-surgery. Primary outcome was feasibility, chosen to provide data to inform design of a successful full-scale RCT. Parameters used for feasibility were recruitment, VLCD adherence, safety, acceptability, attendance and health-related QoL, outlined as follows:

#### Recruitment

Recruitment was measured as the percentage of potential participants contacted who were enrolled (yield) and attrition rate. Attrition was defined as participants who withdrew their consent or failed to attend their post-intervention data collection session. Threshold to meet feasibility criteria was ≥ 35 % yield, based on similar RCT^(24)^, and ≤ 20 % attrition, based on established higher risk of bias at > 20 % attrition^(25)^.

#### Very low calorie diet adherence

Adherence was measured using the VLCD Dietary Adherence Measure (VDAM) (online Supplementary Fig. S1). This tool was custom-designed for the current study by the principal investigator, adapted from the validated Perceived Dietary Adherence Questionnaire for People with Type 2 Diabetes (PDAQ)^(26)^. The VDAM comprised six questions, which asked the extent to which participants had followed the prescription in the previous 14 d, based on intake of: number of VLCD meal replacements, protein-rich foods, high sugar/fat foods, non-starchy vegetables and calorie-free fluids. A higher score indicated better adherence, with a maximum score of 84 indicating 100 % adherence. Scores were collected at each dietitian appointment post-commencement of VLCD for the VLCD group. Threshold to meet feasibility criteria was overall VDAM score of > 42 (> 50 % adherence). This threshold was chosen because the dietary intervention was known (from the previous study^(15)^ and from clinical practice in the VLCD Clinic) to produce clinically significant weight loss with an approximate adherence rate of > 50 %. Additionally, due to the nature of complex dietary interventions, adherence is more prone to fluctuations over time, and this was reflected in the chosen threshold. The VDAM was designed to best reflect adherence to this specific dietary intervention. It was first piloted with several dietitians, and improvements were made based on their feedback prior to use.

#### Safety

Safety was based on LBM due to known increased risk of postoperative complications resulting from preoperative LBM loss^(13,14)^. LBM was measured using bioelectrical impedance analysis via ‘Fresenius Body Composition Monitor’ (Fresenius Medical Care), measured at baseline and post-intervention time points. Thresholds to meet safety criteria were no unfavourable change in body composition (no reduced overall LBM%) for the VLCD group, ≤ 15 % weight loss attributed by LBM (chosen due to studies utilising VLCD with inadequate protein provision resulting in up to 25 % LBM loss^(27)^), plus no serious adverse events or adverse events, as per definitions from government guidelines for safety monitoring and reporting in clinical trials^(28)^. As bioelectrical impedance analysis is highly reproducible and operator-dependent^(29)^, the principal investigator was trained in using the monitor and conducted measurements for all participants.

#### Attendance

Attendance was measured via ‘failure-to-attend’ scheduled data collection (control group) and dietitian appointments (VLCD group) after consenting to participate. Threshold to meet feasibility criteria was ≤ 10 % failure-to-attend rate, in line with acceptable rates for dietitian interventions established by the research site.

#### Acceptability

Acceptability was measured via the AIM (Acceptability of Intervention Measure), a validated tool with strong psychometric properties in similar populations^(30)^. The AIM was distributed to VLCD group via Qualtrics^XM^ (Qualtrics), sent via email at conclusion of last dietitian appointment. The AIM has no ‘cut-off’ score, with higher scores indicating higher acceptability, and instructions for its use suggest creating an average. Threshold to meet feasibility criteria was overall mean score of > 12 (midpoint between minimum 4 and maximum 20).

#### Health-related quality of life

QoL was measured via the Euro-Qual Vertical Visual Analogue Score (EQ-VAS), a measure of overall self-assessed health on a scale of 0–100, which is part of the EQ-5D-3L, a validated tool with good psychometric properties^(31,32)^. It was measured at baseline and post-intervention time points via paper-based survey. Threshold to meet feasibility criteria was no mean/median reduction in VLCD group. The EQ-5D-3L also measures a health state index score, which addresses the five dimensions of mobility, personal care, usual activities, pain/discomfort and anxiety/depression. These were reported as secondary outcomes.

The remaining secondary outcomes included characteristic data, anthropometric data (weight (kg), BMI (kg/m^2^), waist, hip, and neck circumference (cm), waist:hip ratio, and fat mass %,), intervention duration, increase in duration and/or intensity of structured exercise (both groups) or engagement in other dietary interventions (control group), and surgical outcomes. An additional survey, that is, the ‘surgical risk survey’, was designed by the principal investigator using Qualtrics^XM^ (Qualtrics) and piloted by members of the research team prior to distribution to both groups post-consent. The survey questions explored participants’ experience of being informed about their obesity-related surgical risks at the time of surgery booking (both groups), the acceptability of the intervention, and to gather patients’ perspectives on the intervention (intervention group only). There was no scope to validate the survey within this trial. The follow-up period for all participants was 30 d postoperatively to collect surgical outcomes. See online Supplementary Table S2 for surgical outcome definitions and Supplementary Methods for surgical risk survey methodology.

### Sample size and statistical analysis

Due to the lack of previous studies on the same surgical procedures, a sample size calculation was not possible. Therefore, a convenience sample was recruited as feasible within the available time frame at the research site with the aim of at least thirty participants per surgical type (total 90), with reference to attrition rates seen in similar studies^(24)^ and recommendations regarding sample sizes for pilot trials (20 to 80)^(33)^.

All data were analysed using IBM SPSS (IBM Corp. version 29.0). Normality testing was conducted. Between-group differences in continuous data were tested using appropriate non-parametric (Mann–Whitney test) or parametric tests (unpaired Student’s *t* test). Categorical variables were compared using *χ* ^2^/Fisher’s exact tests. Changes between groups were analysed using repeated measures ANOVA. Significance was set at *P* < 0·05. Survey responses were analysed using simple descriptive statistics.

## Results

**Fig. 1.**
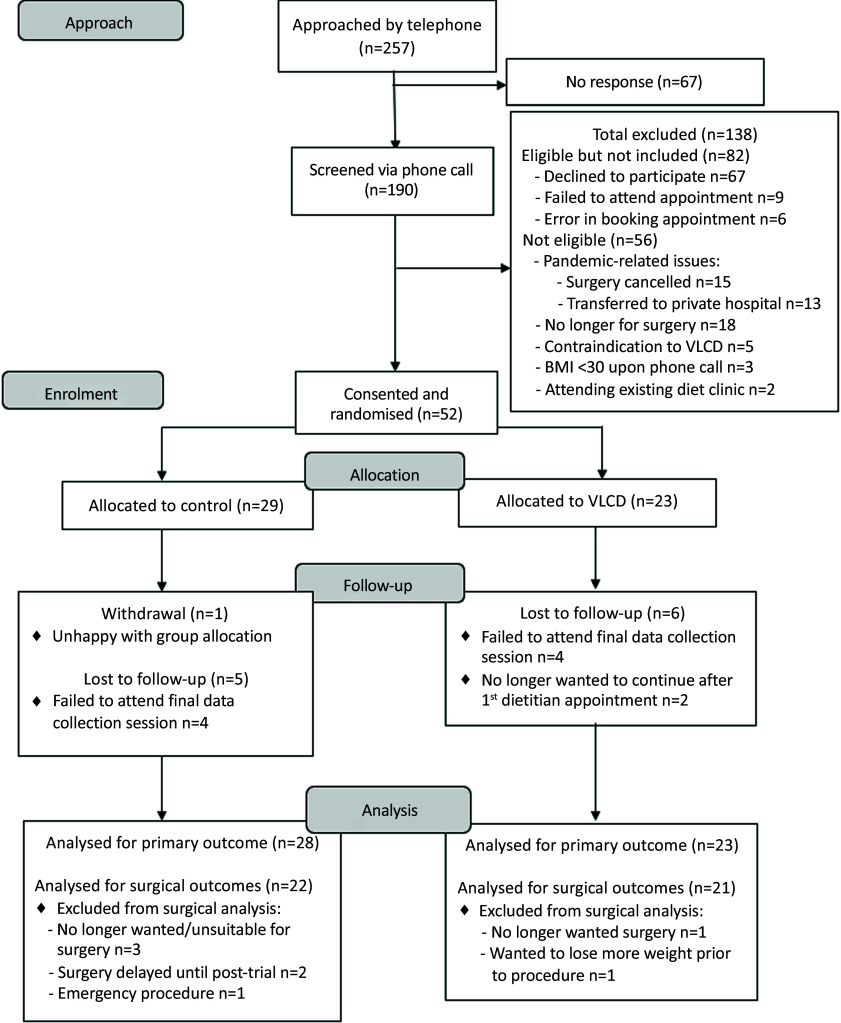
CONSORT diagram – recruitment of participants for the pilot randomised controlled trial.

Participant recruitment is summarised in the CONSORT diagram (Fig. 1). In total, 257 patients were approached by telephone to undertake initial eligibility screening and asked to participate. Sixty-seven patients (26 %) did not respond to the telephone call. After exclusions, fifty-two participants were consented and randomised for inclusion (*n* 23 VLCD and *n* 29 control). One control group participant withdrew consent following randomisation due to discontent with group allocation. There were no consent withdrawals in the intervention group, although two participants advised they did not wish to continue after their first appointment (no reasons provided). One participant’s BMI was < 30 kg/m^2^ (29·2 kg/m^2^) at the time of their first data collection session, but were included as their BMI was ≥30 kg/m^2^ at the time of surgical booking and thus met the eligibility criteria.

### Baseline characteristics

Participant characteristics are summarised in Table 1. VLCD and control groups were similar at baseline, with no statistically significant differences between sex, age (range 22–84 years), BMI or American Society of Anaesthesiologists (ASA) class (*P* > 0·05). Most participants (75 %) had an ASA II (mild systemic disease). BMI ranged from 29·2 kg/m^2^ to 48·6 kg/m^2^, and 16 % had BMI ≥ 40 kg/m^2^. Planned gynaecology procedures included laparoscopic procedure other than hysterectomy (*n* 15), total laparoscopic hysterectomy (*n* 8) and total abdominal hysterectomy (*n* 2). No procedures were planned for the treatment of cancer. A full breakdown of procedures is available in online Supplementary Table S3.

**Table 1.** Participant characteristics recruited to the pilot randomised controlled trial

	Total (*n* 51)		VLCD (*n* 23)		Control (*n* 28)		Between-group *P* value
	*n*	%	*n*	%	*n*	%	
Surgery planned							
Gynaecology	25	49	13	52	12	48	0·450*
Laparoscopic cholecystectomy	18	35	8	35	10	36	
Ventral hernia repair	8	16	2	9	6	21	
Surgical risk factors							
Sex, female	44	86	20	87	24	86	0·900*

	Mean	sd	Mean	sd	Mean	sd	
Age (years)	48	13	47	10	48	15	0·651†
Weight (kg)	100·3	17·3	100·2	19·3	100·4	15·9	0·966†
BMI (kg/m^2^)	35·8	4·6	35·8	4·9	35·8	4·5	0·963†

	*n*	%	*n*	%	*n*	%	
ASA Score I	1	2	1	4	0	0	0·614*
ASA Score II	38	75	16	70	22	79	
ASA Score III	12	24	6	26	6	21	
Hypertension	16	31	6	26	10	36	0·552*
Type 2 diabetes	4	8	1	4	3	13	0·617*
Obstructive sleep apnoea	8	16	3	13	5	18	0·715*
Current smoker	8	16	2	9	6	21	0·441*
Prior abdominal surgery	34	67	14	61	20	71	0·553*

VLCD, very low calorie diet; ASA Score, American Society of Anaesthesiologists Physical Status Classification System^(48)^, in brief; Score I, ‘A normal, healthy patient’; Score II, ‘A patient with mild systemic disease’; Score III, ‘A patient with severe systemic disease’.

Significance = *P* < 0·05.

* Fisher’s exact test.

† Unpaired Student’s *t* test.

### Feasibility outcomes

Threshold criteria for feasibility were met for adherence, safety, attendance and acceptability but not for recruitment, which did not meet thresholds for recruitment or attrition rate (see Table 2). Attrition rate was higher for VLCD group (26 %, *v*. 17 % for control group). Control group %LBM increased by 0·7 ± 2·3 % and VLCD group increased by 2·7 ± 3·8 %, neither of which were statistically significant (*P* > 0·05). No serious adverse or adverse events occurred, and body weight loss attributed by LBM was median 13·3 %. Failure-to-attend rates were similar (6 % in VLCD *v*. 7 % in controls). Six VLCD group participants responded to the AIM survey (26 % response rate) with mean score of 17. Mean health-related QoL significantly increased for both groups: control group by 8·9 ± 15·7 points (*P* = 0·021) and VLCD group by 22·1 ± 15 points (*P* < 0·001).

**Table 2.** Feasibility outcomes for participants in the pilot randomised controlled trial

	Threshold criteria			Result		Satisfied feasibility criteria
Recruitment (both groups)						
Yield*	*n*	≥ 35 %		52		No
	%			20 %		
Attrition†	*n*	≤ 20 %		11		No
	%			21 %		
VLCD adherence (VLCD group only)						
VDAM score‡, % (*n* 18)	Mean	> 42 (> 50 %)		73·6 (88 % adherence)		Yes
	sd			8·2		
Safety (VLCD group only)						
Change in LBM§ (%)	Mean	No overall % reduction		Baseline	32·7	Yes
	sd				7·6	
	Mean			Post	35·4	
	sd				8·5	
Weight loss attributed by LBM§ (%)	Median	≤ 15 %		13·3		Yes
	IQR			0–72		
Serious adverse events/adverse events		No events		Nil		Yes
Attendance (both groups)						
‘Failure-to-attend’ rate		≤ 10 %		7 % (*n* 9/135 appointments)		Yes
Acceptability (VLCD group only)						
AIM score||, (*n* 6)	Mean	> 12		17		Yes
	sd			1·7		
Health-related quality of life (VLCD group only)						
EQ-VAS¶,**	Mean	No reduction		Baseline	56·7	Yes
	sd				18·3	
	Mean			Post	78·8	
	sd				15·3	

VLCD, very low calorie diet; VDAM, VLCD Dietary Adherence Measure; LBM, lean body mass; AIM, Acceptability of Intervention Measure; EQ-VAS, Euro-Qual Vertical Visual Analogue.

* Percentage of potential participants contacted who were enrolled.

† Participants who withdrew their consent or failed to attend their post-intervention data collection session.

‡ Measured using the VDAM adapted for the current study based on a validated tool used for diabetes dietary adherence^(26)^ (≤ 21 = poor adherence, > 45 adequate adherence, > 63 = excellent adherence and 84 = complete adherence).

§ Measured using bioimpedance impedance analysis, via Fresenius Body Composition Monitor, *n* 14 complete pre-post datasets.

|| Measured via the validated AIM^(30)^.

¶ Measured via EQ-VAS within the EQ-5D-3L tool^(31)^, scored out of 100.

** Sixteen complete pre-post datasets.

### Secondary outcomes

#### Duration of treatment

Gynaecology participants received median 54 d (interquartile range (IQR) 13–89) of VLCD/control conditions, laparoscopic cholecystectomy group received median 20 d (IQR 14–26), and hernia repair received 56 d (IQR 46–140). Laparoscopic cholecystectomy time frames were significantly shorter than both gynaecology and hernia repair (Kruskal–Wallis, *P* < 0·001). There was no significant difference between control and VLCD time frames within each surgical group (Kruskal–Wallis: gynaecology *P* = 0·538, LC *P* = 0·721 and hernia repair *P* = 0·739).

#### Anthropometry

Table 3 outlines anthropometric changes from baseline to post-intervention and between groups. Weight loss in the VLCD group reached clinical significance (≥ 5 %). It was also statistically significantly greater in VLCD group (5·5 % *v*. 0·9 %, *P* = 0·004), as was reduction in BMI (–2 kg/m^2^ *v*. −0·2 kg/m^2^, *P* = 0·002), waist circumference (–6·6 cm *v*. +0·6 cm, *P* = 0·004) and hip circumference (–4·9 *v*. −1·8 cm, *P* = 0·031) when compared with controls. In the VLCD group, weight loss attributed to LBM was 0·8 ± 2·5 kg, with a median of 13·3 % (IQR 0–72 %), while weight loss from fat mass was 4·4 ± 5·4 kg, with a median of 58·5 % (IQR 15–77 %). In the control group, weight loss attributed to LBM was 0·4 ± 2·3 kg, with a median of 19·1 % (IQR 0–92 %), and weight loss from fat mass was 0·6 ± 0·9 kg, with a median of 40 % (IQR 0–58 %).

**Table 3.** Anthropometric changes for VLCD and control groups within the pilot randomised controlled trial

	VLCD (*n* 23)				Control (*n* 28)				Change between groups over time				
Outcomes	Baseline		Post		Baseline		Post		*F*	*P*	η_p_^2^	df	Cohen’s d
	Mean	sd	Mean	sd	Mean	sd	Mean	sd					
Weight (kg)*	100·2	19·3	94·7	16	100·4	15·9	99·5	16·9	9·282	0·004	0·171	1	0·891
BMI (kg/m^2^)*	35·8	4·9	33·8	4·3	35·8	4·5	35·6	4·8	10·951	0·002	0·196	1	0·95
Waist circumference (cm)†	114·3	15	107·7	12·5	114	14·3	114·6	17·5	9·818	0·004	0·260	1	1·168
Hip circumference (cm)‡	120·3	11·4	115·4	12·2	122·8	10·8	121	8·7	5·188	0·031	0·161	1	0·689
Neck circumference (cm)§	39	4·2	37·3	5·1	38·5	3·6	38	11·3	4·059	0·053	0·123	1	0·733
Waist:hip ratio‡	0·95	0·095	0·94	0·096	0·93	0·089	0·93	0·91	0·886	0·355	0·033	1	0·329
Fat mass (%)||	50·6	5·9	48·4	6·7	51·8	6·2	51·4	6·4	3·77	0·062	0·112	1	0·692
Lean body mass (%)||	32·7	7·6	35·4	8·5	31·6	9·4	31·3	8·5	1·729	0·198	0·022	1	0·463

VLCD, very low calorie diet; η_p_^2^, partial-eta-squared; df, degrees of freedom.

Significance = *P* < 0·05.

* Complete pre/post datasets: VLCD group: *n* 21, control group: *n* 25.

† Complete pre/post datasets: VLCD group: *n* 12, control group: *n* 18.

‡ Complete pre/post datasets: VLCD group: *n* 12, control group: *n* 17.

§ Complete pre/post datasets: VLCD group: *n* 13, control group: *n* 18.

|| Complete pre/post datasets: VLCD group: *n* 14, control group: *n* 18.

Five control participants (22 %) and five VLCD participants (28 %) reported increased duration and/or intensity of structured exercise, and two control group participants (9 %) reported changing their diet to lose weight from the time of consent.

#### Surgical risk survey and quality-of-life health index scores

Thirty of fifty-one participants responded to the surgical risk survey (59 % response rate – control group (*n* 24) *v*. VLCD (*n* 6)). The full results of the survey are found in online Supplementary Table S4, and the health index score results from the EQ-5D-3L can be found in online Supplementary Results and Supplementary Table S5.

#### Surgical outcomes

Table 4 outlines surgical outcomes for the forty-three participants who underwent elective procedures. Included in the analysis was one VLCD participant whose ovarian cystectomy was abandoned intraoperatively due to dense adhesions and surgical difficulty preventing safe completion. Longest hospital stay was 14 d, for hernia repair within the control group, who experienced conversion from laparoscopic to open procedure, persistent drainage, organ/space surgical site infection and unplanned return to theatre. There were no mortalities and no incidence of any other unfavourable surgical outcomes listed in online Supplementary Table S2.

**Table 4.** Surgical outcomes for VLCD and control groups in the pilot randomised controlled trial

	VLCD (*n* 21)		Control (*n* 22)		*P*
	Median	IQR	Median	IQR	
Anaesthesia induction time (minutes)	20·5	15–36	21	9–57	0·927*
Total operating theatre time (minutes)	112	79–146	104	60–219	0·840*
Operating time (minutes)	86·5	43–118	67	43–188	0·814*
Gynaecology procedures†	117	86–159	127	69–150	0·840*
Laparoscopic cholecystectomy‡	46·5	34–78	54	50–67	0·463*
Hernia repair§	96	96–96	83	54−121	1·0*
Length of hospital stay (days)	1	1–1	1	0·5–1	0·761*

	*n*	%	*n*	%	
Conversion from laparoscopic to open procedure	0	0	1	4·8¶	0·512‡‡
Unplanned injury to other organs	1	4·7	2	9**	1·0
Persistent wound drainage||	N/A		1	50¶	N/A
SSI – superficial	2	10§§	0	0	0·488‡‡
SSI – organ/space	0	0	1	4·8¶	1·0‡‡
Unplanned re-admission	0	0	2	9·5††	0·488‡‡
Unplanned return to emergency department	1	5	1	4·8¶	0·606‡‡
Unplanned return to theatre	0	0	1	4·8¶	1·0‡‡
Estimated blood loss† (gynaecology only)					
‘Minimal’ or ≤ 50 ml	7	64	4	33	0·151||||
100–150 ml	1	13	4	33	
400–500 ml	3	27	0	0	

VLCD, very low calorie diet; IQR, interquartile range; N/A, not applicable; SSI, surgical site infection.

Significance = *P* < 0·05.

* Mann–Whitney test.

† *n* 11 VLCD, *n* 8 control.

‡ *n* 8 VLCD, *n* 7 control.

§ *n* 1 VLCD, *n* 7 control.

|| Nil wound drains placed in VLCD group and two drains placed in control group.

¶ Hernia repair.

** Both lap choles – ‘perforated gallbladder’ and liver injury.

†† Lap chole and hernia repair.

‡‡ Fisher’s exact test.

§§ Both gynaecology procedures.

|||| Fisher–Freeman–Halton exact test.

## Discussion

This pilot study found the RCT design to be feasible to implement in an Australian public hospital setting, by demonstrating adequate VLCD adherence, safety, acceptability, attendance and improved health-related QoL. Surgical outcomes were feasible to collect and will provide preliminary data to assist in designing future full-scale RCT. Recruitment yield and attrition rates were significantly impacted by the active COVID-19 pandemic, leading to missing data and smaller sample sizes for analysis. Thus, the authors concluded that, with adjustments to recruitment strategy and data collection methods to mitigate hospital disruptions, a full-scale trial using this design is feasible.

Studies have shown significant levels of hospital avoidance in relation to the increase in cases of COVID-19 in the community^(34)^. Given this trial was being undertaken during a community outbreak of the highly infectious Omicron strain, causing cessation of healthcare services including elective surgery, it is prudent to conclude that this influenced attrition and attendance rates in the current study. Twenty per cent of participants who were able to be contacted were ineligible due to COVID-19-related issues, and attritions were mostly participants who failed to attend their last appointment for data collection. The use of a more valuable financial incentives to attend all appointments capturing participants on the day of their surgery and/or utilising technology to facilitate off-site data collection may minimise attrition rates.

VLCD adherence comfortably exceeded the feasibility threshold, indicating a high adherence to dietitian-led treatment. This echoes non-surgical studies which show that structured VLCD protocols provided by dietitians are largely well adhered to and are well tolerated^(35,36)^. Dietary adherence is particularly difficult to measure accurately due to the high chance of bias through self-reporting methods such as food diaries, which have been used in similar studies^(18)^. Measuring urinary ketones has been done in other studies, but weight loss can occur irrespective of presence of ketones and as such, we chose not to utilise this method.

Despite clinically significant weight loss, LBM made up less than 15 % of total body weight lost in the VLCD group. Non-surgical studies on VLCD where the protein provision is standardised for all participants (e.g. 45 g protein/d) and therefore likely inadequate for some participants show that up to 25 % of total body weight loss experienced is LBM^(37)^, although these were extreme in their calorie restriction (< 600 kcal/d), potentially influencing results. Interestingly, recent research indicates that the combination of protein supplementation and exercise training could enhance the preservation of LBM while undergoing VLCD^(38)^. We hypothesise the preservation of LBM in our trial may have been assisted by individualised dietary protein prescriptions by the dietitian, monitoring protein intake over time. Although caution should be taken when interpreting the LBM results in the current study, ours is the second RCT to show acceptable changes to LBM% via individualised protein prescription and monitoring using dietitian-led VLCD for non-bariatric elective surgery patients^(24)^. Neither study included patients undergoing cancer treatment or major gastrointestinal surgery, who pose higher risk of poor surgical outcomes through underlying catabolic and malabsorption processes, regardless of BMI^(39,40)^. Therefore, safety cannot be confirmed for other procedures.

This is the first RCT to examine preoperative weight loss for gynaecology surgery patients. This is surprising, considering excess visceral and pelvic adipose tissue can make port and view of critical structures more difficult^(41)^, with longer operative times and increased risk of 30-d perioperative complications^(42)^. Although there are no gynaecology studies with which to compare our results, a recent study on forty-three males awaiting prostatectomy found a preoperative low-calorie diet and exercise programme helped reduce pelvic fat mass and blood pressure^(43)^. Other smaller observational studies have shown a significant reduction in visceral and pelvic fat resulting from VLCD^(44,45)^ but did not examine surgical outcomes. However, there is high potential for dietitian-led VLCD intervention to be used as a preoperative optimisation strategy for procedures accessing organs within the pelvis, with wide-reaching potential to improve outcomes for the high proportion of endometrial cancer patients living with obesity who require surgery, given that over 50 % of all endometrial cancers are attributable to obesity^(46)^.

### Limitations and learnings for future trials

There were several limitations in this study. The cohort in this trial were of relatively lower ‘risk’ than bariatric surgery studies (mean BMI 35·8 kg/m^2^ with maximum limited to 48·6 kg/m^2^, and majority with only mild systemic disease), and this may have influenced outcomes. Eligible participants were already deemed ‘ready for surgery’ as they were recruited from surgical waiting lists, and as such it may be that patients with higher BMI were not placed on surgical waiting lists due to their weight, therefore taking them out of the pool of patients to recruit from. Recruitment methods should be adjusted in future trials to recruit in person from surgical outpatient clinics rather than from waiting lists.

The Fresenius Body Composition Monitor was used to measure LBM via bioelectrical impedance analysis in this trial, which was not validated for this cohort, and more often used to measure fluid mass. Utilisation of DEXA or CT scan should be prioritised for measuring LBM in future trials to enhance validity. Additionally, the VDAM tool was custom-designed and piloted for use in this trial as no validated tools existed to measure VLCD adherence. Validating the VDAM prior to this trial would have been ideal, but it was outside the study scope. For future full-size trials, it should be formally tested and validated.

Specifics of physical activity data were not recorded, which potentially could have confounded results. Despite this, similar proportions in each group reported increased intensity and/or duration of physical activity. While the control group was free to undertake alternative weight loss strategies, only two individuals reported having done so. These results provide reassurance that the weight loss in VLCD group can be attributed to the dietitian-led intervention. Future trials should record duration, intensity and frequency of physical activity.

Utilisation of an individualised dietary intervention conducted in a real-world setting meant that there were several potential confounders that were not measured or controlled for in the present trial. These included different meal replacement products used, number of dietitian appointments, VLCD prescription, for example, Phase 1, Phase 2, and how many participants required additional protein to meet protein requirements. To look at these components individually was outside the scope of this trial, but our preliminary study^(15)^ showed most participants followed the most calorie-restricted programme that fits the true VLCD definition (Phase 1) for the majority of treatment time, and it was likely this was replicated in this trial. Further, when we measured duration on treatment or control conditions, there was no significant difference between groups. Literature shows that weight loss is more dependent on duration of VLCD than level of calorie restriction^(47)^, and as such we can suppose the confounding differences between diet prescriptions may have been limited in this way. In saying this, the frequency of dietitian support through fortnightly appointments for the VLCD group likely enhanced weight loss efforts. The control received less contact, and thus this potentially influenced their behaviours. Controlling for time frames and standardising the number of appointments provided were difficult to achieve in the current trial, given the extent of COVID-19 delays and the inflexible nature of elective surgical services within a large public hospital. Additionally, no data were collected on reasons participants did not attend their second data collection sessions, and this may have helped interpret our feasibility outcomes. In future trials, number of appointments should aim to be standardised to reduce potential bias, prescription and intake of diet composition should be measured, and a validated VLCD adherence tool should be utilised.

The small number of respondents who completed the AIM (*n* 6) make the results difficult to evaluate and interpret. Furthermore, participant satisfaction with the intervention may have motivated a biased response rate. Difficulties in recruitment and electronic survey distribution are likely reasons for small sample size. A paper-based survey given and collected immediately after appointment(s) would likely have yielded more responses. Despite this, attendance of 93 % to dietitian appointments, improvement in health-related QoL and significant weight loss signify the intervention likely had high acceptability.

Given the small sizes of the subgroups of surgical procedures in the current study, it is not surprising that statistically significant between-group differences could not be demonstrated. In large public hospitals in Australia, procedures can be performed by numerous surgeons, and thus surgical outcomes may have been affected by the level of surgeons’ skill and training, and blinding of surgeons was not possible to mitigate for unconscious bias. Despite this, it is encouraging that outcome trends seemed to favour the VLCD group and highlight the need for larger sample sizes to determine the VLCD Clinic’s efficacy. In future trials, controlling for confounding factors such as performing surgeon, use of surgical safety protocols and blinding of surgeons should be prioritised where possible.

### Conclusion

This pilot RCT examined the feasibility of implementing a full-scale RCT in a public hospital setting to evaluate the effect of a preoperative dietitian-led VLCD Clinic intervention for adults living with obesity awaiting elective gynaecology and general surgery and marks the first RCT exploration of preoperative weight loss for gynaecology surgery patients. The current study design was deemed feasible, but recruitment and data collection methods should be adjusted in the design of a full-scale trial to mitigate likely disruptions to conducting research in real-world clinical settings. Pleasingly, other feasibility thresholds of adherence, safety, acceptability, attendance and health-related QoL were met, and weight, waist and hip circumference were significantly reduced in the VLCD group. Although this pilot was underpowered, 30-d postoperative complications were more common in the control group. The results provide convincing evidence to support prehabilitation models which utilise dietitian-led VLCD, in the climate of ever-increasing demand to operate on patients living with obesity and associated co-morbidities.

## Supporting information

Griffin et al. supplementary material Griffin et al. supplementary material

## Supplementary material

For supplementary material/s referred to in this article, please visit https://doi.org/10.1017/S0007114524000114
