# Supplementary material for: Preoperative dietitian-led Very Low Calorie Diet (VLCD) Clinic for adults living with obesity undergoing gynaecology, laparoscopic cholecystectomy and hernia repair procedures: a pilot parallel randomised controlled trial
Source: Br J Nutr. 2024 Jan 15;131(8):1436–46. doi: 10.1017/S0007114524000114 (PMC10950445; doi:10.1017/S0007114524000114)
Supplement: Griffin et al. supplementary material [file S0007114524000114sup001.docx]

**Supplementary Materials – Index**

| **Supplementary Figures** | |
| --- | --- |
| Supplementary Figure S1 - VLCD Dietary Adherence Tool (VDAM) | Pg 2 |
| **Supplementary Methods** | |
| Surgical outcome data collection and Surgical Risk Survey Methodology including full survey | Pg 3-6 |
| **Supplementary Tables** | |
| Supplementary Table S1 – Nutritional breakdown of VLCD meal replacement products | Pg 7 |
| Supplementary Table S2 - Surgical outcome definitions | Pg 8-9 |
| Supplementary Table S3 - Breakdown of participants by surgery procedure | Pg 10 |
| Supplementary Table S4 - Responses to Surgical Risk survey | Pg 11-13 |
| Supplementary Table S5 - Quality of life scores per health index | Pg 14 |
| **Supplementary Results** | |
| Survey outcomes from EQ-5D-3L | Pg 15 |

**Figure S1 – VLCD Dietary Adherence Measure Tool**

**
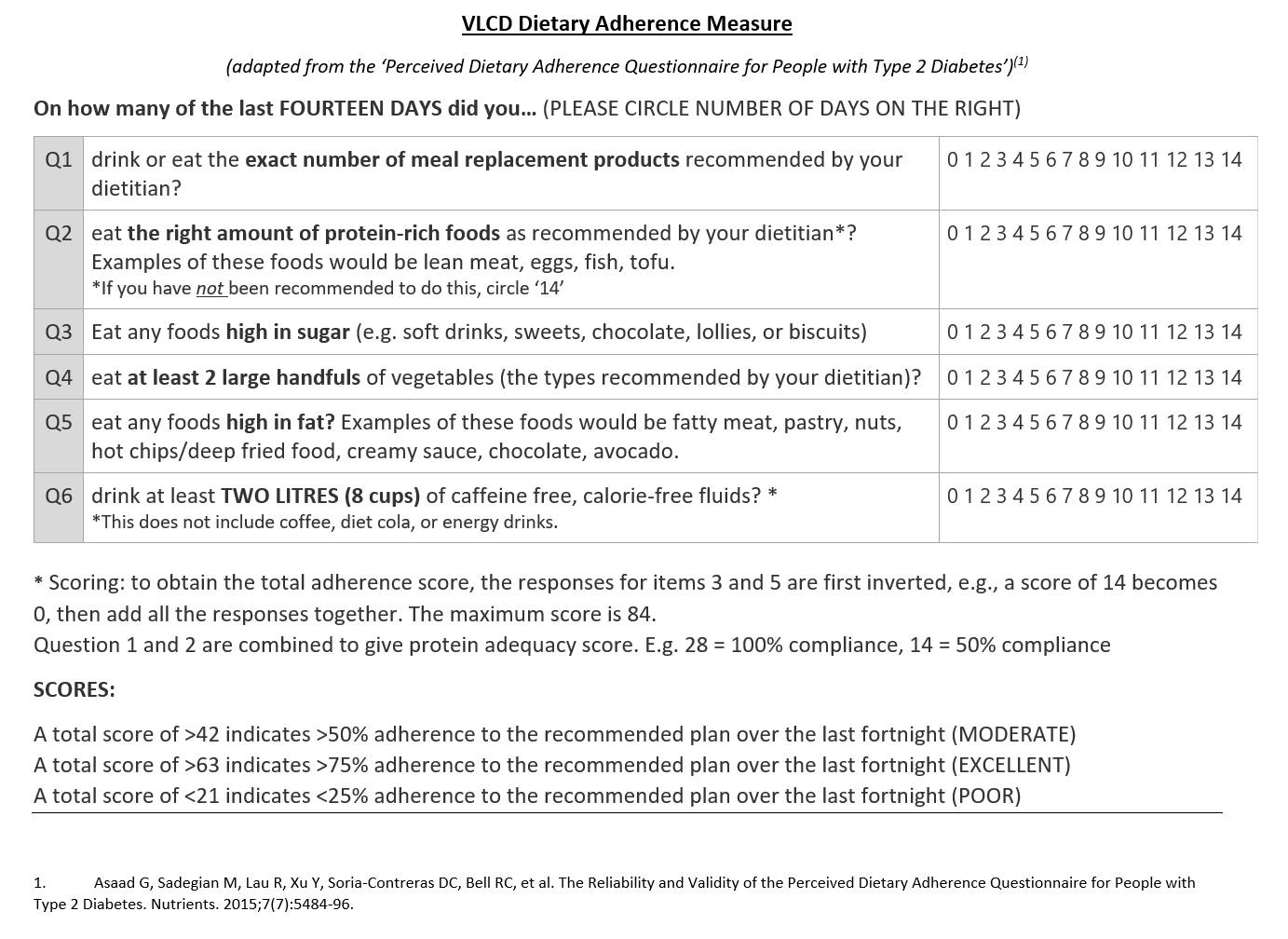
**

**Supplementary Methods**

**Surgical Risk Survey Methodology**

The survey questions were based on the theoretical framework of acceptability developed by Sekhon et al^(1)^. The closed-ended questions used a 5-point Likert scale, which asked to rate agreement with statements based on domains of: Affective Attitude (satisfaction with intervention), Burden (effort required), Ethicality (if it aligns with their values), Intervention Coherence (how it is intended to work), Opportunity Costs (financial), Perceived Effectiveness, and Self-efficacy (confidence in being able to participate). Other closed ended-questions related to participants’ knowledge of obesity-related perioperative risks and whether this influenced dietary/exercise behaviour, preference for intervention modality (in person vs. telehealth) and postoperative dietary follow-up, and suggestions for improving the intervention. The three open-ended questions asked for qualitative responses: what they liked about the intervention, suggestions for improvement, and other feedback.

**Surgical Outcome Methodology**

Surgical outcomes collected were based on ACS NSQIP (American College of Surgeons National Surgical Quality Improvement Program), a standardised approach used internationally to measure relevant postoperative outcomes up to 30 days postoperatively^(2)^. Table 1 outlines the surgical outcomes and the collection methodology used, adapted from the NSQIP, combined with the definitions from the publicly available ACS Surgical Risk Calculator^(3)^ and based on operational practices at the research site. Any unclear surgical outcome was discussed with a local surgical nurse trained in NSQIP data collection for final decision.

**Surgical Risk Survey**

Q1 What type of surgery are you receiving?

- Laparoscopic Cholecystectomy (getting my gallbladder removed) (1)
- Gynaecological surgery (e.g. hysterectomy, ovaries removed etc) (2)
- Hernia repair (3)
- I'm not sure / none of the above (4)

Q2 Which surgeon are you under the care of, for this surgery?

- Dr Mahi Ranasinghe (1)
- Dr Peita Webb (2)
- Dr Peter Wysocki (3)
- Dr Ian Bloomfield (4)
- Dr Teong Chuah (5)
- Dr Hastika Ellepola (6)
- Dr Huba Brezovsky (7)
- Dr Janet Draper (8)
- Dr Darsana Boban (9)
- Dr Yogapragasen Gounden (10)
- Dr Shaffy Kochar (11)
- Dr Leela Sharma (12)
- I am not sure (13)

Q3 Did somebody at Logan Hospital talk to you about the **risks of surgery being increased due to your weight** before or at the time you signed the paperwork for your surgery?

- Yes (1)
- No (2)
- I don't know / I can't remember (3)

*Skip To: Q6 If Q3 = No*

*Skip To: Q6 If Q3 = I don't know / I can't remember*

Q4 Who was the person who talked to you about this?
(choose more than one if more than one person talked to you about this in relation to your current surgery)

1. My surgeon (1)
2. Nurse (2)
3. Other health professional or person at the hospital (e.g. Physio, other doctor at the hospital) - please type here who it was: (3) ________________________________________________

Q5 Did this conversation make you **change your behaviour, physical activity, or what you ate** to try to lose weight?

- Yes (1)
- No (2)
- I don't know / I can't remember (3)

Q6 Please indicate how much you agree / disagree with the following statements:

|  | Completely disagree (1) | Disagree (2) | Neither agree nor disagree (3) | Agree (4) | Completely agree (5) |
| --- | --- | --- | --- | --- | --- |
| Losing weight will make my surgery safer (1) |  |  |  |  |  |
| I am motivated to lose weight before my surgery (2) |  |  |  |  |  |

Q7 Which arm of the study (trial) do you belong to?

- Group A: Measurements done at two time-points before surgery (Standard Approach) (1)
- Group B: Fortnightly dietitian appointments leading up to surgery (Fit for Surgery Approach) (2)
- I don't know / I can't remember (3)

*Skip To: End of Survey If Q7 = Group A: Measurements done at two time-points before surgery (Standard Approach)*

*Skip To: End of Survey If Q7 = I don't know / I can't remember*

STATEMENT For the remaining questions, ‘the program’ refers to the appointments with the dietitian, the diet plan recommended to you and the advice and support given by the dietitian at Logan Hospital leading up to your surgery.

Q8 Please indicate how much you agree / disagree with the following statements:

|  | Completely disagree (1) | Disagree (2) | Neither agree nor disagree (3) | Agree (4) | Completely agree (5) |
| --- | --- | --- | --- | --- | --- |
| I **understand what is involved in the program** that I have been asked to follow prior to my surgery (1) |  |  |  |  |  |
| I **feel positive about the program** that I am engaged in prior to my surgery (2) |  |  |  |  |  |
| I **feel stressed about the program** that I am engaged in prior to my surgery (3) |  |  |  |  |  |

Q9 Please indicate how much you agree / disagree with the following statements:

|  | Completely disagree (1) | Disagree (2) | Neither agree nor disagree (3) | Agree (4) | Completely agree (5) |
| --- | --- | --- | --- | --- | --- |
| The **program will require a lot of effort** for me to complete (1) |  |  |  |  |  |
| The program is **too expensive** (2) |  |  |  |  |  |
| The program **takes me a lot of time** (3) |  |  |  |  |  |

Q10 Please indicate how much you agree / disagree with the following statements:

|  | Completely disagree (1) | Disagree (2) | Neither agree nor disagree (3) | Agree (4) | Completely agree (5) |
| --- | --- | --- | --- | --- | --- |
| Before I started this program, **I was worried about what I had to do** in order to be successful on this program (1) |  |  |  |  |  |
| I now **feel confident to follow the advice** given to me within the program (2) |  |  |  |  |  |
| I believe **this program will be effective** in reducing the chance of things going wrong during and after my surgery (such as infections, excess bleeding, and a longer stay in hospital) (3) |  |  |  |  |  |

Q11 Please indicate how much you agree / disagree with the following statements:

|  | Completely disagree (1) | Disagree (2) | Neither agree nor disagree (3) | Agree (4) | Completely agree (5) |
| --- | --- | --- | --- | --- | --- |
| The program **fits well with my ethical values** (in other words, the program is in line with the things I personally value in life) (1) |  |  |  |  |  |
| I would **recommend this program** to someone I care about (2) |  |  |  |  |  |

Q12 Would you like to receive support for weight loss or weight maintenance after your surgery?

- Yes (1)
- No (2)
- I'm not sure (3)

*Skip To: Q13 If Q12 = Yes*

Q13 How would you like to receive support for weight loss or weight maintenance after surgery? (tick all that apply)

1. I would prefer to seek support from my GP (1)
2. I would like information on community weight loss support groups I could access (2)
3. I would like a referral to a private dietitian at my own costs or via Medicare, if eligible (3)
4. Other support option (please write here) (4) ________________________________________________

Q14 This study may lead to the program being offered to more patients prior to their surgery. When answering the following questions, **please consider ways to improve the program for the future**.
Your opinion is valuable to us!

Q15 What is your preferred method of attending the dietitian appointments for this program? (tick all that apply)

1. Face to Face (in person appointments) (1)
2. Telehealth (Video call) (2)
3. Telephone (3)

Q16 Which aspect, or aspects of the program did you **like**?

________________________________________________________________

Q17 What suggestions do you have to **improve** the program?

________________________________________________________________

Q18 Do you have any other thoughts, comments or feedback regarding this program? Please enter them here.

________________________________________________________________

**Supplementary Table S1 – Nutritional breakdown of Optifast and Optislim products**

| Nutrient | Average of three products per day (average of all types including soups, bars, desserts, and shakes) | |
| --- | --- | --- |
|  | **Optifast** | **Optislim** |
| Energy | 2610kJ (621kcal) | 2432kJ (579kcal) |
| Protein | 59g | 55g |
| Carbohydrate | 64g | 60g |
| Fibre | 12g | 0-3g |
| Primary protein source | Animal | Vegetable |
| Based on Recommended Daily Intake (RDI) or Adequate Intake (AI) for Women 31-50 years ^α^ | **100% RDI or AI met with three products?** | |
|  | **Optifast** | **Optislim** |
| Iron | YES | YES |
| Vitamin A | YES | YES |
| Vitamin B1 (Thiamine) | YES | YES |
| Vitamin B2 (Riboflavin) | YES | YES |
| Vitamin B3 (Niacin) | YES | YES |
| Vitamin C | YES | YES |
| Vitamin D | YES | YES |
| Vitamin E | YES | YES |
| Vitamin K | YES | NO (does not contain Vit K) |
| Folic Acid | NO (83% met) | YES |
| Vitamin B12 | YES | YES |
| Vitamin B6 | YES | YES |
| Calcium | YES | NO (70% met) |
| Zinc | YES | YES |
| Potassium | YES | NO (71% met) |
| Iodine | YES | YES |
| Selenium | YES | NO (85% met) |
| VLCD characteristics | **Optifast** | **Optislim** |
| Variety of products available and number of flavours | Total: 14  *Flavours:* 7 shakes, 3 bars, 2 soups, 2 desserts | Total: 14  *Flavours:* 6 shakes, 5 bars  3 soups |
| Average cost per product (based on RRP)  ^β^ | $5.10 | $3.40 |
| Gluten Free | Shakes: Yes  Desserts: Yes  Bars/Soups: No | No |
| Lactose Free | Shakes: No  Desserts: No  Soups: No  Bars: <1g lactose per bar | Shakes: No  Bars: unknown  Soups: unknown |

*RRP = Recommended Retail Price ^α^ based on Nutrient References Values via National Health and Medical Research Council ^(4)^*  ^β^ *Prices based on Recommended Retail Price at Chemist Warehouse, Amcal Chemist or brands’ websites, as per products available as at 7^th^ October 2023*

**Supplementary Table S2 - Surgical outcome definitions and methodology used to collect from randomised controlled trial participants’ electronic medical chart.**

| **Intraoperative outcomes** | |
| --- | --- |
| Operating time | The earlier time of either the specific surgical preparation of the participant or commencement of the skin preparation until the time all instrument and sponge counts have been completed and verified as correct; all dressings and drains are secured; and the surgical team had completed all procedure related activities on the participant. |
| Anaesthesia induction time | Anaesthetic induction time was time from preparation of anaesthetic induction to start of operating time. |
| Total operating room time | ‘Wheels in Wheels Out’ – the time the participant directly wheeled into the operating theatre and wheeled out of the operating theatre. |
| Laparoscopic converted to open procedure | Unplanned conversion during procedure |
| Estimated blood loss during operation (Gynaecology procedures only) | Measured in millilitres (mL), documented by surgeon |
| **Postoperative outcomes (up to 30 days)** | |
| Mortality | Death |
| Myocardial infarction (MI) | Including ‘Non-STEMI’ or ‘NSTEMI’ |
| Requirement for intensive care bed | Yes/No, number of days |
| Requirement for rehabilitation | Admission or transfer to rehab ward or sub-acute facility |
| Unplanned return to operating theatre | Any reoperations for any reason after the planned procedure which was not planned prior to or at that time |
| Unplanned re-admission to hospital | For any reason that crosses at least two midnights, with no prior documentation that this readmission was part of a plan of care |
| Unplanned presentation to emergency department | With no prior documentation that the presentation was part of a plan of care |
| Unplanned intubation | Re-intubated at any time after their surgery whether they were/were not intubated for surgery |
| Requirement for blood transfusion | Excluding blood initiated prior to the surgical start time and continuing intraoperatively and/or postoperatively, excluding transfusions of other blood products |
| Surgical Site Infection (superficial, deep, or organ/space) | e.g., ‘SSI’, ‘site infection’, ‘infected surgical wound’, ‘wound abscess’, etc.  *Superficial:* involves only skin or subcutaneous tissue of the incision/sites integral to the procedure  *Deep:* involves deep soft tissues  *Organ/Space*: involves any of the anatomy (e.g., organs or spaces), other than the incision, which was opened or manipulated during procedure |
| Wound disruption | A loss of the integrity of fascial closure or whatever closure was performed in the absence of fascial closure |
| Pneumonia | Hospital Acquired Pneumonia, e.g., ‘HAP’ |
| On ventilator 48 hours | If patient removed from theatre ventilated, start time is from leaving theatre |
| Kidney failure requiring dialysis | Not on regular dialysis preoperatively |
| Stroke / Cerebral Vascular accident | Includes TIA (Transient Ischaemic Attack) |
| Venous Thromboembolism / Pulmonary Embolism | New diagnosis of venous thrombosis (superficial or deep) confirmed by imaging e.g., MRI |
| Urinary Tract / Acute Kidney Infection | Examples include ‘UTI’, ‘AKI’ |
| Hospital acquired pressure injury | Stage 2 or above only, documented by wound care nurse |
| Persistent wound drainage | Continued fluid extrusion from the operative site occurring beyond 72 hours postoperatively |
| Unplanned injury to other abdominal organs during surgery | Including bile duct injury for laparoscopic cholecystectomy |
| Length of stay | Number of admitted days crossing midnight |

**Supplementary Table S3: breakdown of participants by surgery type and procedure**

| **Surgery Type and Procedure** | **Total, n (%)**  **(n=51)** | **VLCD, n (%)**  **(n=23)** | **Control, n (%)**  **(n=28)** |
| --- | --- | --- | --- |
| **Gynaecology** | 25 (49) | 13 (52) | 12 (48) |
| Other laparoscopic procedure | 15 (60) | 9 (69) | 6 (50) |
| Total laparoscopic hysterectomy | 8 (32) | 3 (38) | 5 (63) |
| TLH alone | 2 (25) | 1(33) | 1 (20) |
| TLH + bilateral salpingectomy/tubal ligation | 6 (75) | 2 (66) | 4 (80) |
| Total abdominal hysterectomy | 2 (16) | 1 (8) | 1 (8) |
| **Laparoscopic cholecystectomy** | 18 (35) | 8 (35) | 10 (36) |
| **Hernia Repair** | 8 (16) | 2 (9) | 6 (21) |
| Primary repair | 6 (75) | 2 (100) | 4 (67) |
| Secondary repair | 2 (25) | 0 (0) | 2 (33) |

Note: the % add up to 100% across the rows and not down the columns

**Table S4: Responses to Surgical Risk Survey**

|  | | **Laparoscopic cholecystectomy (n=8)**  n (%) | | **Gynae**  **(n=15)**  n (%) | **Hernia**  **(n=7)**  n (%) | **Total**  n (%) |
| --- | --- | --- | --- | --- | --- | --- |
| **Risks of obesity vs surgery explained (total n=30)** | | | | | |  |
| Yes | | 4 (50) | | 9 (60) | 2 (29) | 15 (50) |
| No | | 4 (50) | | 6 (40) | 4 (57) | 14 (47) |
| I don’t know / I can’t remember | | 0 | | 0 | 1 (14) | 1 (3) |
| **Person who explained this (total n=16)** | | | | | | |
| Surgeon | | 2 (40) | | 7 (78) | 2 (100) | 11 (69) |
| Nurse | | 1 (20) | | 2 (22) | 0 | 3 (19) |
| Other Health professional (n=1 text: anaesthetist) | | 2 (40) | | 0 | 0 | 2 (13) |
| **Change in diet/physical activity behaviours due to risk explanation (total n=15)** | | | | | | |
| Yes | | 2 (50) | | 8 (89) | 1 (50) | 11 (73) |
| No | | 2 (50) | | 1 (11) | 1 (50) | 4 (27) |
| **Losing weight will make my surgery safer (total n=30)** | | | | | | |
| Completely disagree | | 0 | | 0 | 0 | 0 |
| Disagree | | 0 | | 0 | 0 | 0 |
| Neither agree nor disagree | | 0 | | 4 (27) | 0 | 4 (13) |
| Agree | | 3 (38) | | 4 (27) | 7 (100) | 14 (47) |
| Completely agree | | 5 (63) | | 7 (47) | 0 | 12 (40) |
| **I am motivated to lose weight before my surgery (total n=30)** | | | | | | |
| Completely disagree | | 0 | | 0 | 0 | 0 |
| Disagree | | 1 (13) | | 0 | 0 | 1 (3) |
| Neither agree nor disagree | | 0 | | 2 (13) | 1 (14) | 3 (10) |
| Agree | | 4 (50) | | 7 (47) | 3 (43) | 14 (47) |
| Completely agree | | 3 (38) | | 6 (40) | 3 (43) | 12 (40) |
| **VLCD Group only (n=6) responses to questions regarding VLCD intervention** | | | | | | |
|  | | **Laparoscopic cholecystectomy (n=0)** | | **Gynae (n=5)** | **Hernia (n=1)** | **Total**  **(n=6)** |
| **I understand what is involved in the program that I have been asked to follow** | | | | | | |
| Agree | | 0 | | 2 | 1 | 3 |
| Completely agree | | 0 | | 3 | 0 | 3 |
| **I feel positive about the program that I am engaged in prior to my surgery** | | | | | | |
| Agree | | 0 | | 2 | 1 | 3 |
| Completely agree | | 0 | | 3 | 0 | 3 |
| **I feel stressed about the program that I am engaged in prior to my surgery** | | | | | | |
| Completely disagree | | 0 | | 1 | 0 | 1 |
| Disagree | | 0 | | 4 | 1 | 5 |
| **The program will require a lot of effort for me to complete** | | | | | | |
| Neither agree nor disagree | | 0 | | 2 | 0 | 2 |
| Agree | | 0 | | 2 | 1 | 3 |
| Completely agree | | 0 | | 1 | 0 | 1 |
| **The program is too expensive** | | | | | | |
| Disagree | | 0 | | 3 | 1 | 4 |
| Neither agree nor disagree | | 0 | | 2 | 0 | 2 |
| **The program takes me a lot of time** | | | | | | |
| Disagree | | 0 | | 4 | 1 | 5 |
| Neither agree nor disagree | | 0 | | 1 | 0 | 1 |
| **Before I started this program, I was worried about what I had to do to be successful** | | | | | | |
| Neither agree nor disagree | | 0 | | 3 | 0 | 3 |
| Agree | | 0 | | 2 | 1 | 3 |
| **I now feel confident to follow the advice given to me within the program** | | | | | | |
| Agree | | 0 | | 3 | 1 | 4 |
| Completely agree | | 0 | | 2 | 0 | 2 |
| **I believe this program will be effective in reducing the chance of things going wrong during and after my surgery** | | | | | | |
| Completely disagree | | 0 | | 1 | 0 | 1 |
| Neither agree nor disagree | | 0 | | 1 | 0 | 1 |
| Agree | | 0 | | 2 | 1 | 3 |
| Completely agree | | 0 | | 1 | 0 | 1 |
| **The program fits well with my ethical values** | | | | | | |
| Neither agree nor disagree | | 0 | | 1 | 0 | 1 |
| Agree | | 0 | | 3 | 1 | 4 |
| Completely agree | | 0 | | 1 | 0 | 1 |
| **I would recommend this program to someone I care about** | | | | | | |
| Agree | | 0 | | 3 | 1 | 4 |
| Completely agree | | 0 | | 2 | 0 | 2 |
| **Would you like to receive support for weight loss or weight maintenance after surgery?** | | | | | | |
| Yes | 0 | | 3 | | 1 | 4 |
| No | 0 | | 2 | | 0 | 2 |
| I’m not sure | 0 | | 0 | | 0 | 0 |
| **How would you like to receive support for weight loss/ maintenance after surgery?** | | | | | | |
| I would prefer to seek support from my GP | 0 | | 2 | | 1 | 3 |
| I would like information on community weight loss support groups I could access | 0 | | 0 | | 0 | 0 |
| I would like a referral to a private dietitian at my own costs or via Medicare, if eligible | 0 | | 1 | | 0 | 1 |
| Other:  ‘I will continue with this program on my own’ n=1  ‘I am continuing with a low GI diet’ n=1 | 0 | | 2 | | 0 | 2 |
| **What is your preferred method of attending the dietitian appointments?** | | | | | | |
| Face to face | 0 | | 5 | | 1 | 6 |
| Telehealth video call | 0 | | 0 | | 0 | 0 |
| Telephone | 0 | | 0 | | 0 | 0 |
| F2F + Telehealth | 0 | | 0 | | 0 | 0 |
| F2F + Telephone | 0 | | 0 | | 0 | 0 |
| Telehealth + Telephone | 0 | | 0 | | 0 | 0 |
| **Open-ended question responses** | | | | | | |
| **“Which aspect/s of the program did you like?”** | | | | | | |
| I felt the dietitian was there to help and support me, lots of good information given so I understood the aims and expected outcomes without feeling overly pressured | | | | | | |
| Consultations visits check ins | | | | | | |
| Knowing there was support | | | | | | |
| The support and guidance I got from the dietitian | | | | | | |
| The encouragement with having appointments, variety of food, learning more about foods and what they contain | | | | | | |
| **“What suggestions do you have to improve the program?”** | | | | | | |
| Not really sure I feel everything is covered quite well | | | | | | |
| Nil | | | | | | |
| I think the program is good | | | | | | |
| It all good | | | | | | |
| Perhaps an exercise program | | | | | | |
| **“Do you have any other thoughts, comments or feedback regarding this program?”** | | | | | | |
| I think this is a great tool and would be beneficial to patients going forward. Thank you for allowing me to participate | | | | | | |
| Nil | | | | | | |
| Excellent research programme | | | | | | |
| None | | | | | | |
| I found it most beneficial and made me realise what bad habits I had. It has changed my way of thinking about food | | | | | | |

**Supplementary Table S5: Quality of life scores per Health Index**

|  | **Control n (%)** | | **VLCD n (%)** | |
| --- | --- | --- | --- | --- |
|  | **Pre**  **(n=28)** | **Post**  **(n=23)** | **Pre**  **(n=23)** | **Post**  **(n=16)** |
| **Mobility** | | | | |
| 1 (No problems) | 18 (64) | 16 (70) | 16 (70) | 11 (69) |
| 2 (Some problems) | 10 (36) | 7 (30) | 7 (30) | 5 (31) |
| 3 (Bed-bound) | 0 | 0 | 0 | 0 |
| **Personal Care** | | | | |
| 1 (No problems) | 25 (89) | 20 (87) | 19 (83) | 14 (88) |
| 2 (Some problems) | 3 (13) | 3 (13) | 3 (13) | 2 (13) |
| 3 (Unable) | 0 | 0 | 1 (4) | 0 |
| **Usual Activities** | | | | |
| 1 (No problems) | 17 (61) | 15 (65) | 13 (57) | 11 (69) |
| 2 (Some problems) | 10 (36) | 8 (35) | 10 (43) | 5 (31) |
| 3 (Unable to do) | 1 (3) | 0 | 0 | 0 |
| **Pain** | | | | |
| 1 (None) | 10 (36) | 11 (48) | 3 (13) | 8 (50) |
| 2 (Moderate) | 16 (57) | 12 (52) | 19 (83) | 7 (44) |
| 3 (Extreme) | 2 (7) | 0 | 1 (4) | 1 (6) |
| **Anxiety/Depression** | | | | |
| 1 (None) | 16 (57) | 15 (65) | 11 (48) | 9 (56) |
| 2 (Moderate) | 12 (43) | 8 (35) | 9 (48) | 5 (31) |
| 3 (Severe) | 0 | 0 | 3 (13) | 2 (13) |
| **Cumulative Health Index scores (total of five domains of mobility, personal care, usual activities, pain, and anxiety/depression)** | | | | |
| **Group** | | **Pre** | **Post** | ***p*^α^** |
| VLCD, median (range) | | 7 (5-11) | 7 (5-10) | .020 |
| Control, median (range) | | 7 (5-12) | 7 (5-10) | .020 |

May not add up to 100% due to rounding.

*α* Wilcoxon Rank test, score is out of possible range of 5 to 15, 5= best Quality of life, 15 =worst quality of life)

**Supplementary Results**

*Surgical risk survey and Health Index Quality of Life outcomes*

All 51 participants were emailed the survey and 30 responded (59% response rate, n=24 control, n=6 VLCD). Gynaecology was most represented (50%, 15/30). Half indicated that the risks of surgery relating to obesity had been explained to them at surgery booking (50% n=15/30), most of these reported this had prompted a change to diet/exercise (73%, n=11/15), and most agreed that losing weight would make their surgery safer and reported motivation to lose weight because of this (87%, n=26/30).

Questions relating to acceptability, asked of the n=6 VLCD participants showed 100% agreed that they understood what was involved in the intervention, felt positive about it, now felt confident to follow the advice, and would recommend the intervention to someone they care about. Most agreed that the intervention fit well with their values and reported they would like to receive support for weight loss/maintenance post-surgery, and the intervention was affordable (n=4/6). None reported feeling stressed about the intervention, and most disagreed that it took lot of time and effort (n=5/6). The preferred dietitian appointment modality was face-to-face appointments (n=6, 100%).

**References**

1. Sekhon M, Cartwright M, Francis JJ (2017) Acceptability of healthcare interventions: an overview of reviews and development of a theoretical framework. *BMC Health Services Research* **17**, 88.

2. American College of Surgeons (2021) ACS National Surgical Quality Improvement Program. <https://www.facs.org/quality-programs/acs-nsqip> (accessed March 1st 2023)

3. Bilimoria KY, Liu Y, Paruch JL *et al.* (2013) Development and evaluation of the universal ACS NSQIP surgical risk calculator: a decision aid and informed consent tool for patients and surgeons. *J Am Coll Surg* **217**, 833-842.e831-833.

4. National Health & Medical Research Council (NHMRC) (2017) Nutrients. <https://www.nrv.gov.au/nutrients> (accessed 18th September, 2019
